# Monitoring diaphragm function in a patient with myasthenia gravis: electrical activity of the diaphragm vs. maximal inspiratory pressure

**DOI:** 10.1186/s40560-017-0262-8

**Published:** 2017-11-28

**Authors:** Yukiko Koyama, Takeshi Yoshida, Akinori Uchiyama, Yuji Fujino

**Affiliations:** 1 Department of Anesthesiology and Intensive Care Medicine, Osaka University Graduate School of Medicine, 2-15, Yamadaoka, Suita, Osaka, 565-0871 Japan; 2 Physiology and Experimental Medicine, Departments of Critical Care Medicine and Anesthesia, Hospital for Sick Children, University of Toronto, 686 Bay Street, Toronto, ON M5G 0A4 Canada

**Keywords:** Myasthenia gravis, Electrical activity, Diaphragmatic weakness, Mechanical ventilation, Weaning

## Abstract

**Background:**

Maximal inspiratory pressure (MIP) is used to assess respiratory muscle strength of patients with myasthenia gravis (MG) requiring ventilatory support. Electrical activity of the diaphragm (E-di) has been used to guide weaning.

**Case presentation:**

The MIP and tidal volume/ΔE-di (the patient-to-ventilator breath contribution) were monitored in a 12-year-old girl with MG requiring ventilator support. The same ventilatory settings were maintained until extubation. During weaning, MIP increased slightly, but varied unpredictably. Tidal volume/ΔE-di decreased at a constant rate as muscle strength recovered.

**Conclusion:**

In this patient with muscle weakness, E-di was a reliable tool to monitor weaning from mechanical ventilation.

## Background

Myasthenia gravis (MG) affects primary neuromuscular transmission, is characterized by skeletal muscle weakness and fatigue, and can involve respiratory muscles [1, 2]. Some patients with MG experience diaphragmatic weakness [2] and breathing difficulty that may require mechanical ventilator support. Evaluation of respiratory muscle strength is an important part of the weaning process.

Maximal inspiratory pressure (MIP) is used to assess respiratory muscle function [1]. It is noninvasive, easy to measure, and cost effective, but has very low specificity and reproducibility [3]. MIP has been found to vary from day to day, with the investigator performing the test, and with patient cooperation [3]. The electrical activity of the diaphragm (E-di) can be measured with commercially available nasogastric probes that are used to control the ventilator and decrease the dys-synchrony between spontaneous and mechanical breaths [4]. Because E-di reflects neural activity, it may change with recovery of respiratory muscle function earlier than other clinical variables such as MIP, tidal volume, respiratory rate, gas exchange, and physical signs. E-di might thus be a better assessment of diaphragm function during the clinical course of MG patients receiving ventilator support. The clinical usefulness of continuous recording of E-di to monitor the recovery of diaphragmatic function was compared with MIP in a 12-year-old MG patient over a period of 8 days. The patient and parents described in this case report reviewed the manuscript and provided their written informed consent for its publication. The presentation was approved by the Institutional Ethics Committee of Osaka University Graduate School of Medicine (approval number 13311). It was an observational, not an interventional, study.

## Case presentation

A 12-year-old girl 141 cm tall and weighing 26.4 kg presented with severe MG. The patient was healthy at birth, and in May 2013, she reported a history of persistent low-grade fever, blepharoptosis, weakness of the extremities, and dysarthrosis. In July 2013, seropositivity for anti-acetylcholine receptor (Ach-R) antibody, a positive Teng Shillong test, and surface electromyogram findings led to a diagnosis of whole-body MG. Despite the administration of immunoglobulin, difficulty in swallowing persisted and dyspnea progressed. At the end of July, she was intubated for mechanical ventilatory support and transferred to our institution. The initial ventilator settings were pressure-controlled, with a fractional inspired oxygen concentration of 0.35, respiratory rate of 10 breaths/min, inspiratory pressure (above positive end-expiratory pressure) of 10 cmH_2_O adjusted not to exceed a tidal volume of 10 ml/kg, and a positive end-expiratory pressure (PEEP) of 5 cmH_2_O. The PaO_2_/FiO_2_ ratio was > 300 mmHg, chest X-rays showed no abnormalities and signs of pneumonia. The patient was sedated with midazolam, fentanyl, and dexmedetomidine, but conscious and oriented (Richmond Agitation-Sedation Scale 0).

**Fig. 1:**
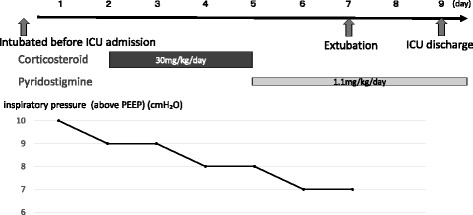
The clinical course of the patient in the ICU. The medications are as described

Respiratory muscle strength was evaluated every morning by MIP and diaphragm activity in relation to tidal volume (*V*_T_/ΔE-di). E-di was measured with a size-appropriate multiple-array esophageal electrode (E-di catheter, 12 Fr, 125 cm) that was inserted using a method like that used to insert an ordinary nasogastric tube and positioned following the manufacturer’s recommendations. ΔE-di was calculated as the maximum minus the minimum E-di during inspiration [5]. Sedative drugs were discontinued at least 30 min before measuring MIP and *V*_T_/ΔE-di, and the patient was fully conscious when the measurements were started. The measurements were stopped if at least one the following occurred. The blood oxygen saturation (SpO_2_) was < 90%, the respiratory rate was > 30/min, the systolic blood pressure was ≥ 180 mmHg, or cardiac arrhythmias developed. MIP was measured three times. The patient was encouraged to perform a maximal inspiratory effort with airway occlusion toward a closed circuit, and the largest test value was recorded. *V*_T_/ΔE-di was measured with the same respirator settings, i.e., pressure support ventilation (PSV) mode, PEEP of 5 cmH_2_O, and pressure support of 5 cmH_2_O. *V*_T_/ΔE-di was not obtained on day 6 for technical reasons. A few minutes after measuring MIP, the ventilator was set to PSV mode. After breathing was stabilized, airway pressure, flow, and E-di were recorded for 2 min. E-di measurements, acquired via the Servo-i ventilator, were transferred to a personal computer using Servo-i tracker software ® (Maquet, Critical Care AB, Sweden) for the entire duration of the study. Sedatives were restarted after completion of the measurements. On day 2 in the intensive care unit (ICU), the first of three large, consecutive daily doses of corticosteroid (methylprednisolone, 30 mg/kg/day) was given, and pyridostigmine bromide (1.1 mg/kg/day) was started on day 5. The patient’s clinical course is summarized in Fig. 1.

**Fig. 2:**
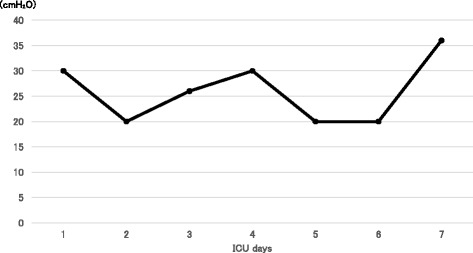
Maximal inspiratory pressure during the clinical course. The values varied day-by-day, not showing a consistent trend

**Fig. 3:**
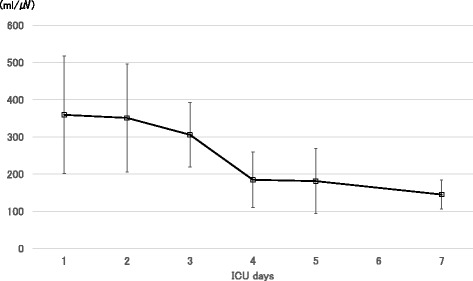
The ratio of tidal volume to electrical activity of the diaphragm (*V*_T_/ΔE-di). *V*_T_/ΔE-di decreased consistently over time. The value on day 6 is missing because of technical reasons

Figure 2 shows the course of MIP. It increased slightly over time, but changed unpredictably day-by-day. MIP increased during high-dose corticosteroid therapy, but then suddenly decreased afterward. *V*_T_/ΔE-di was measured by the same method every day (Fig. 3). In contrast to the unpredictable time course of MIP, *V*_T_/ΔE-di decreased consistently after day 4, which was the last day of corticosteroid pulse therapy. On day 7, the patient passed a spontaneous breathing trial (SBT) with PSV, 5 cmH_2_O PEEP and 5 cmH_2_O pressure support for 30 min. Taking her clinical symptoms into account, her diaphragmatic strength appeared to have recovered sufficiently, and she was extubated on that day. Hemodynamic instability, signs of respiratory muscle fatigue, dyspnea, airway obstruction, or decrease in arterial oxygen saturation were not observed following extubation. The patient was discharged from the ICU on day 9.

## Discussion and conclusions

This case demonstrated that continuous recording of *V*_T_/ΔE-di may potentially be a better, more reliable indicator of the recovery of diaphragmatic function in MG patients than MIP. Respiratory failure requiring mechanical ventilation is a severe complication of MG [1] caused by respiratory muscle weakness. MIP, which is frequently used to assess the strength of respiratory muscles [1], relies on investigator interpretation and the patient’s voluntary efforts. Consequently, MIP results are not always reproducible in mechanically ventilated patients [3]. In this patient, continual recording of MIP failed to show the recovery of diaphragmatic function in the present case and was not useful for indicating when to extubate. Efficacy of Edi should be useful, especially in the management of uncooperative patients such as children.

Recent evidence indicates that E-di-derived indices may be helpful predictors of weaning outcome [5–7], especially with continuous monitoring, which can help to compensate for individual variability of E-di and helps to promptly detect pathophysiological changes. The findings in this patient are consistent with those of Wolf et al. [5], who also used *V*_T_/ΔE-di to assess extubation readiness in pediatric patients and observed a decreased *V*_T_/ΔE-di ratio in patients on PSV who passed a spontaneous breathing test. The finding that *V*_T_/ΔE-di decreased consistently as diaphragmatic function recovered after corticosteroid pulse therapy suggests that patients who can generate higher diaphragmatic activity relative to *V*_T_ may have better preserved diaphragmatic function. *V*_T_/ΔE-di may thus be useful in assessing diaphragmatic function during an SBT in settings other than the clinical course of neuromuscular disease. On the other hand, Barwing et al. [7] found an early reduction in the *V*_T_/ΔE-di ratio in adult patients who failed an SBT proved difficult to wean. The *V*_T_/ΔE-di was reduced in patients who failed the SBT mainly because of a steep increase in E-di, which was explained by a strong effort to maintain sufficient tidal ventilation. The differences in the findings of these two studies might be explained by the duration of the SBT (30 min vs. 1 h) and the age difference of the study populations. Young children have a different respiratory pattern from adults because of less developed muscle strength.

The SBT procedure differs with ventilator settings such as PSV, continuous positive airway pressure (CPAP), and T-piece (T-tube). Ferguson et al. [8] and Nascimento et al. [9] reported that the SBT did not predict extubation failure in pediatric patients on PCV because high pressure support with small endotracheal tubes overestimated the readiness for extubation. The most common cause of extubation failure was inadequate gas exchange in the lower respiratory tract problem, which suggested that performing the SBT with pressure support caused respiratory insufficiency. Khemani et al. [10] showed that the breathing effort of children with a CPAP of 5 cmH_2_O was similar to the breathing effort after extubation and that pressure support during extubation readiness tests significantly underestimated postextubation breathing effort. Esteban and co-workers [11] reported that the percentage of adult patients successfully extubated after SBT was higher with PSV than with T tubes. The percentages of patients reintubated or successfully extubated after failing the SBT were not different. PSV was associated with higher SBT success rates, but not to higher risk for reintubation. Some patients ventilated with a T-tube system failed the SBT because of respiratory load, but were successfully extubated when the overload was eliminated by pressure support. Cabello et al. [12] found that in a population of difficult-to-wean patients, PSV plus PEEP significantly modified the breathing pattern, inspiratory muscle effort, and cardiovascular response compared with a T-piece system. As in other studies, it was possible that excessive support in PSV and the need for muscle strength in T-piece systems affected SBT performance. We thought that the differences about *V*_T_/ΔE-di ratio were influenced by the method of SBT.

Previous studies reported a correlation between the intensity of the diaphragmatic electromyogram and transdiaphragmatic pressure [13, 14], which implies that the E-di can be useful to evaluate inspiratory effort during recovery from MG. The *V*_T_/ΔE-di ratio may be influenced by increased resistive load, decreased pulmonary or chest wall compliance, mechanical alterations of diaphragm function, and reduced neuromuscular coupling [15]. The *V*_T_/ΔE-di ratio is also influenced by age because of respiratory mechanics and lung and inspiratory muscle function [16]. Recent studies differ in patient population and study design, but agree that the *V*_T_/ΔE-di ratio may be useful in assessing diaphragm function [5–7]. As the ratio can be obtained independent of investigator judgment ant patient cooperation, it would be especially helpful in the pediatric population. It is difficult to draw any general conclusions about the predictability of *V*_T_/ΔE-di for extubation based on the findings in this patient. The daily changes in MIP and *V*_T_/ΔE-di were measured in the absence of known absolute cutoff values.

The study limitations included not measuring neuromuscular drive, which was evaluated as the decrease in airway opening pressure at 0.1 s (P_0.1_) after the onset of an inspiratory effort against an occlusion. Neuromuscular effort was evaluated by the decrease in P_0.1_. However, a significant correlation of ΔE-di and P_0.1_ has been shown in critically ill children [5]. A second limitation was the limited size of the catheters as four types of catheters were available for patients of adult to infant ages. This patient was young, and a catheter of appropriate size was available. If she had been much younger, an appropriately sized catheter would not have been available. Third, special equipment and catheters are needed to measure E-di, which adds to the cost of care. We conclude that E-di monitoring was a benefit in this patient with MG, effectively evaluating respiratory muscle strength. This method warrants further comparison to MIP for management of ventilator weaning.

## Abbreviations

E-di: Electrical activity of the diaphragm
MG: Myasthenia gravis
MIP: Maximal inspiratory pressure
*V*_T_: Tidal volume
